# Thermoresponsive Hydrogels and Their Biomedical Applications: Special Insight into Their Applications in Textile Based Transdermal Therapy

**DOI:** 10.3390/polym10050480

**Published:** 2018-04-27

**Authors:** Sudipta Chatterjee, Patrick Chi-leung Hui, Chi-wai Kan

**Affiliations:** Institute of Textiles and Clothing, The Hong Kong Polytechnic University, Hung Hom, Hong Kong, China; sudipta.chatterjee@polyu.edu.hk (S.C.); Kan.chi.wai@polyu.edu.hk (C.-w.K.)

**Keywords:** thermoresponsive hydrogel, natural polymer, biomedical application, drug delivery, transdermal therapy, textile

## Abstract

Various natural and synthetic polymers are capable of showing thermoresponsive properties and their hydrogels are finding a wide range of biomedical applications including drug delivery, tissue engineering and wound healing. Thermoresponsive hydrogels use temperature as external stimulus to show sol-gel transition and most of the thermoresponsive polymers can form hydrogels around body temperature. The availability of natural thermoresponsive polymers and multiple preparation methods of synthetic polymers, simple preparation method and high functionality of thermoresponsive hydrogels offer many advantages for developing drug delivery systems based on thermoresponsive hydrogels. In textile field applications of thermoresponsive hydrogels, textile based transdermal therapy is currently being applied using drug loaded thermoresponsive hydrogels. The current review focuses on the preparation, physico-chemical properties and various biomedical applications of thermoresponsive hydrogels based on natural and synthetic polymers and especially, their applications in developing functionalized textiles for transdermal therapies. Finally, future prospects of dual responsive (pH/temperature) hydrogels made by these polymers for textile based transdermal treatments are mentioned in this review.

## 1. Introduction

Hydrogels made of hydrophilic components are porous, three-dimensional, interpenetrating, polymeric network containing large amounts of water or biological fluids without losing their structure at physiological temperature and pH [1,2,3]. The ability of hydrogels to hold water comes from hydrophilic functional groups of hydrophilic polymers, while their resistance to dissolution arises from cross-linking between polymeric chain networks. Hydrogels can be classified as natural, synthetic and hybrid, depending on the nature of polymers. Hydrogels are generally stabilized by molecular entanglements and other forces like hydrogen bonding, ionic and hydrophobic interactions and hydrogels can be chemically crosslinked by covalent bonds [4,5]. The dissolutions of hydrogels are done by changing external conditions such as temperature, pH and ionic strength. High affinity for water gives hydrogels the physical properties resembling living tissues, such as compactness and low interfacial tension with aqueous media [6]. Hydrogels are extremely suitable for a wide variety of bio-medical applications because of their high water content and the possible control over the swelling kinetics [7,8,9,10,11,12]. The self-healing and in situ forming hydrogels are very important from biomedical point of view as it can provide a means for simple, “custom-made diagnostics” [13,14]. A polymer solution can be prepared and allowed to gel in situ, after chemical/ionic crosslinking, photopolymerization, ionic crosslinking, or in response to physical stimuli such as temperature [15,16,17,18]. Chemical and biochemical stimuli include pH, ionic strength and molecular recognition events [19,20,21]. 

Various polymers are capable of forming hydrogels and depending on their unique biological and physiochemical functions hydrogels provide a wide variety of applications [22,23,24,25,26]. Hydrogels can be prepared from both natural and synthetic polymers. Instead of insufficient mechanical strength of natural polymer based hydrogels, they offer several advantageous properties such as biodegradability, biocompatibility and biologically recognizable moieties which support cellular activities [27], while synthetic polymer hydrogels do not possess such bioactive properties but they have well-defined structures which can be modified to yield tailored degradability and functionality [28]. The diversity of polymeric hydrogels provides a variety of potential benefits for biomedical applications in particular [1,29,30,31,32,33,34]. These properties make hydrogels highly biocompatible and applicable for developing highly effective drug delivery system (DDS) [35,36,37,38,39,40]. Apart from drug delivery applications, the hydrogels are applied for tissue engineering, surface coating, bone healing, developing contact lenses and several diagnostics tools [41,42,43,44,45,46,47,48]. Nowadays, textile applications of hydrogels are found in the form of drug delivery systems where transdermal drug delivery was successfully achieved [49,50] and thermoresponsive hydrogels in particular are effectively forming DDS for textile applications [51,52,53,54,55]. Thermoresponsive hydrogels from various natural and synthetic polymers find a wide range of biomedical applications such as drug delivery, cell encapsulation, tissue repair, wound dressing and others [56,57,58,59,60,61,62]. 

Thermoresponsive polymers are capable of rapidly changing their physical properties with increasing temperature and they belong to the class of stimuli-responsive materials [63,64]. Materials exhibiting a phase transition in response to change in external conditions such as temperature, pH, ionic strength and electric currents are known as “stimuli-responsive” [5]. The ‘stimuli-responsive’ materials are very important from a biomedical point of view as these materials are sensitive to temperature and/or pH change of the surroundings. A critical solution temperature (CST) may be defined as a temperature at which the polymer solution undergoes separation from one phase to two phases [5]. Thermoresponsive polymers remain as liquid at low temperature and form semi-solid gel at higher temperatures and these polymers exhibit a volume phase transition at a certain temperature during gelling which causes a sol-gel transition [15,65]. Thermoresponsive polymers with lower critical solution temperature (LCST) become insoluble upon heating and polymers with upper critical solution temperature (UCST) become soluble upon heating [66]. In the formation of injectable thermoresponsive hydrogels, the process exploits the capability of polymers to form gels in situ at body temperature condition [67,68,69]. The gelation of thermoresponsive polymers is entropy driven and the negative free energy of thermogelation indicates a thermodynamically favorable process [70,71,72]. The thermogelation process is reversible and the gels can return to solution phase after heat used as stimulus is removed [73,74,75]. Thermosensitive polymers maintain hydrophobic–hydrophilic balance in their structure and a minute temperature increase around their critical solution temperature (LCST) result in collapsing of polymers chains in order to adjust the hydrophobic and hydrophilic interactions between the polymer chains and the aqueous medium [76].

**Figure 1:**
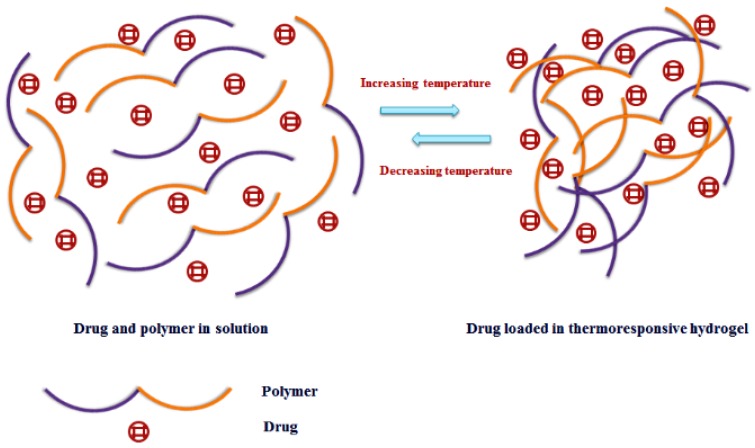
Thermoresponsive hydrogel formation using temperature as a trigger.

**Figure 2:**
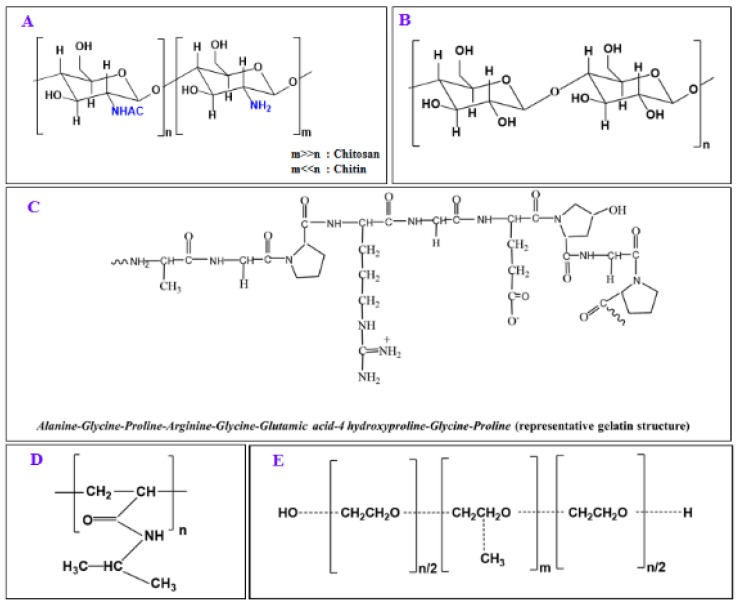
Chemical structure of chitosan (**A**); cellulose (**B**); gelatin (**C**); pNIPAAm (**D**); PF127 (**E**).

This review article encompasses various thermoresponsive natural and synthetic polymers which are capable of forming thermoresponsive hydrogels, nanoparticles and membranes. The formation of thermoresponsive hydrogel from the polymers using temperature change as a trigger is represented in Figure 1. The review article mainly focuses on thermoresponsive hydrogels which are having intense biomedical applications especially in drug delivery for cancer therapy, transdermal drug therapy used in smart textiles, tissue repair and bone regeneration [1,2,5]. The source, structure, properties and biomedical applications of natural thermoresponsive polymers, including chitosan, cellulose, gelatin and synthetic thermoresponsive polymers like poly(*N*-isopropylacrylamide) (pNIPAAm), Pluronics^®^ or Poloxamers namely pluronic F127 (PF127), are discussed in this review. The chemical structures of chitosan (A), cellulose (B), gelatin (C), pNIPAAm (D) and PF127 (E) are given in Figure 2. Several chemical modifications on the polymeric backbone of such compounds are included in the review to discuss and overcome the shortcomings of thermogels with the polymers alone. Some other natural polymers, such as xyloglucan, dextran, poly(γ-glutamate) and elastin and elastin like polypeptide/oligopeptide and synthetic polymers such as polyoxazoline, poly(organophosphazenes), poly(ethylene glycol)/biodegradable polyester copolymers, can form thermoresponsive hydrogels, which find versatile biomedical applications especially for developing drug delivery systems [15]. Nevertheless, the further discussion in the review has been focused on the thermoresponsive hydrogels of five polymers (three natural polymers namely chitosan, cellulose and gelatin and two synthetic polymers pNIPAAm and PF127) to highlight their applications in textile based transdermal treatment and recent advancement of dual responsive (pH/temperature) hydrogels using these polymers for suitable applications in textiles especially for transdermal therapy.

## 2. Natural Polymers

### 2.1. Chitosan

Chitosan, the deacetylated product of chitin, is used in a wide range of biomedical applications. Chitin is commercially available from exoskeleton of crustacean and chitin being mostly insoluble in all solvents, finds better applications after deacetylation in the form of chitosan which is soluble in dilute aqueous acids. Chitosan, a natural cationic biopolymer made of glucosamine units (and traces of *N*-acetyl glucosamine) can form hydrogels with most desirable hydrogel structures as this polymer is hydrophilic, biocompatible, biodegradable and furthermore, degradable via human enzymes. The chemical structure of chitosan was given in Figure 2. Chitosan hydrogels are prepared in different shapes such as beads, films, liquid gels, powders, textile fibers, capsules, microspheres, microparticles and sponges [77,78]. In all form of chitosan hydrogels, the compound is either physically associated or chemically cross-linked to form the hydrogel. Tripolyphosphate (TPP)/chitosan hydrogel beads, prepared under coagulation conditions at 4 °C in the presence of gelatin, were used for the controlled and sustained release of drugs [79]. The polyelectrolyte hydrogels of chitosan and polyvinyl pyrrolidone, made by irradiation, were applied as a pH sensitive delivery system for bovine serum albumin [80]. The hydrogels made of chitosan and poly acrylic acid by graft copolymerization, using *N*,*N*′-methylene-bis-(acrylamide) as a cross-linker, were applied for colon specific drug delivery and the hydrogels showed great potential for their application in oral colon-specific drug delivery systems [81]. Chitosan hydrogel cross-linked with glutaraldehyde was tested in a breast cancer xenograft mouse model and the hydrogel loaded with ^131^I-norcholesterol showed a reduction in the progression rate of the tumor and prevented 69% of tumor recurrence and metastatic spread [82].

Chitosan based thermoresponsive hydrogels were made after combining with other polymers having intrinsic thermoresponsive properties. Chitosan-pluronic (CP) thermoresponsive hydrogel was formed by grafting pluronic onto chitosan using carbodiimide chemistry and the hydrogel was designed as an injectable cell delivery carrier for cartilage regeneration [83]. The CP solution showed a sol-gel transition around 25 °C with the storage modulus (G’) of 104 Pa, highlighting the potential of this material as an injectable scaffold for cartilage regeneration. The reported sol-gel transition of CP thermoresponsive hydrogel around 25 °C was well below the normal body temperature of 37 °C [83]. Poly(ethylene glycol) grafted chitosan based thermoreversible hydrogels showed sustained release of drugs and the gelation was found to be possible in physiological pH values [84]. However, PEG grafting on chitosan could not be exceeded by 36 wt % and it became non-injectable between 36 and 45 wt %, as excess PEG grafting would suppress hydrophobic interactions between chitosan chains and result in a solution that did not gel at body temperature [84]. The drug delivery vehicles based on thermosensitive hydrogels using chitosan, hyaluronic acid and *N*-isopropylacrylamide (NIPAAm), showed controlled release of the analgesic drug nalbuphine in vitro [85]. The possible drawback of the system is that the hydrogels had gelation temperatures which were well below body temperature and thus, they could readily became gels after injection into the body [85]. A thermosensitive hydrogel developed from the copolymer of NIPAAm and water-soluble chitosan was applied for chondrogenic differentiation of human mesenchymal stem cells (hMSC) in a minimally invasive manner through a simple injection [86]. Moreover, the sol-gel transition of the thermosensitive was perfect for in vivo *applications* because the LCST was 32 °C [86]. The thermosensitive hydrogel made from chitosan and αβ-glycerophosphate showed sustained release of drugs (adriamycin and 6-mercaptopurine) [87]. The thermoresponsive hydrogel was better projected as delivery system for hydrophilic drugs than hydrophobic drugs as the release of hydrophilic drug adriamycin followed more sustained manner than that of hydrophobic drug 6-mercaptopurine [87]. A chitosan-glycerophosphate salt (GP) thermoresponsive hydrogel was applied for neural tissue engineering and it showed having good biocompatibility and sustained release property [88]. It showed sol-gel transition at physiolocal pH (around pH 7.0) and a body temperature of 37 °C. Moreover, the mechanism of gelation did not involve any covalent cross-linker, organic solvent and detergent [88]. The double syringe method was applied to form poloxamer gel combined with chitosan and sodium tripolyphospahte (TPP) and the gel was formed in situ from poloxamer-TPP and poloxamer-chitosan solutions in two syringes [89]. The injectable in situ hydrogel of carboxymethyl chitosan and oxidized alginate loaded with curcumin was used for a skin wound healing application [90]. In the study method [90], nano-curcumin with improved oxidative stability was used for a better dermal wound healing process [90]. Carboxymethyl chitosan modified pluronic gels were applied for localized delivery of paclitaxel and paclitaxel micelles were dispersed in a pluronic thermogel system, which was crosslinked with carboxymethyl chitosan by glutaraldehyde [91]. The hydrogels possessed lower viscosity, higher swelling ratio, stronger mechanical property and longer term drug release with promising drug delivery system for hydrophobic drug in cancer therapy [91]. The modified pluronic thermogel system significantly increased the water solubility of paclitaxel to 1000-fold with the drug loading content about 40% and also, increased the mechanical strength of the thermoresponsive gel. Furthermore, retention time of the drug was extended at the tumor sites [91].

Chitosan based hydrogels were applied in textile industry and, the chitosan based hydrogels are of special interest in designing “smart” textile materials for biomedical applications especially for the treatment of atopic dermatitis (AD) [92]. In clinical practice, a textile therapy was developed based on chitosan based hydrogel system using microencapsulation technology for treatment of AD [93,94]. The Chinese herbal medicines (PentaHerbs and cortex moutan) were successfully loaded into chitosan and sodium alginate composite microcapsules using an emulsion chemical crosslinking method and a pad-dry-cure approach was then employed to coat the microcapsules onto the surface of cotton fabrics. The resultant microcapsules were fully evaluated in terms of surface morphology, particle size distribution, in vitro drug release behavior and cytotoxicity study and the drug loaded textiles showed excellent controlled drug release properties, with a release duration of seven days [93,94]. Nevertheless, the average particle size values of microcapsules loaded with Cortex Moutan (3.8 µm) [93] and PentaHerbs (7.4 µm) [94] were quite big and any delivery system bigger than 1 µm was considered not very suitable for transdermal therapy or any other drug delivery application. The hydrogel film composed of chitosan and honey was developed for textile applications, as it is capable of water vapor transmission and water absorption [95]. The film satisfied the strength and elongation requirements of the wound dressing and higher zone of inhibition against *Staphylococcus aureus* and *Escherichia coli* [95]. The hydrogel film also satisfied other requirements of wound dressings like thickness, weight, folding endurance and degradation. But, no drug delivery application was shown for the system and the mechanisms of water absorption and water vapor transmission were not clearly explained. The other textile applications of chitosan included development of chitosan composite hydrogel based textile scaffolds for tissue engineering and the scaffolds were prepared by a freeze-drying technique using woven PES fabric with well-defined macro porosity coated with a biodegradable chitosan-collagen membrane. The system provided a three-dimensional structure for cell attachment and growth [96,97]. 

### 2.2. Cellulose

**Figure 3:**
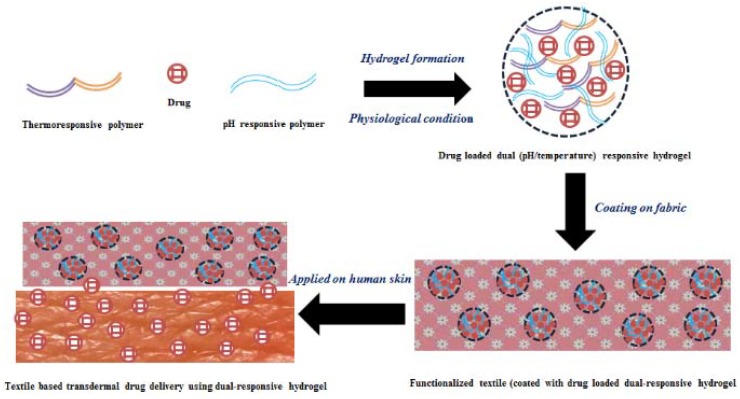
Textile based transdermal drug delivery using dual (pH/temperature) responsive hydrogel.

Cellulose is the most abundant renewable resource on earth and this natural polymer is made up of long chains of glucose molecules, chemically bonded together. Cellulose is an important structural component of the primary cell wall of green plants and many varieties of algae and thereby, fruits and vegetables are excellent sources of cellulose. A wide range of new functional materials from cellulose are being developed because of the increasing demand for environmentally friendly and biocompatible products such as cellulose [98]. Cellulose contains abundant hydroxyl groups which are used to prepare hydrogels having a wide range of biomedical applications. The chemical structure of cellulose was given in Figure 3. Hydrogels derived from cellulose can be prepared by the crosslinking of aqueous solutions of methylcellulose, ethylcellulose, sodium carboxymethylcellulose and hydroxypropyl methylcellulose and cellulose based-hydrogels are used for tissue engineering, drug delivery and other biomedical applications [99,100]. Nevertheless, the main limitation of cellulose based hydrogels is its low solubility in both water and most organic solvents due to the hydrogen-bonded structure [101]. Nanocellulose is a nanostructured material comprising of cellulose nanocrystals (CNC) that can display interesting effects on the gelation mechanism of hydrogels, can improve mechanical and dimensional stability and are capable of favoring drug release [102,103]. Cellulose nanofibers (NFC) also belong to the nanocellulose class and NFCs are used to form cellulose based hydrogels by suspending NFC fibrils by way of a simple mechanical treatment [104]. NFCs are used in a wide range of drug delivery applications [100] and the addition of molecules such as hemicellulose can provide additional benefits such as anticancer and antioxidative properties and can also increase the mechanical stability of the materials [105,106].

Methylcellulose (MC) can form thermoresponsive hydrogel with sol-gel transition in the temperature range of 60–80 °C and can change into solution upon cooling [107]. Gelation procedure for MC involves three steps namely, hydrophobic association, phase separation and gelation. Various grades of MC show different molecular weights (number average) and these vary in the range 10,000–220,000 Da. The physical properties of MC, such as solubility are affected by the degree of substitution. Commercial MC is a heterogeneous polymer consisting of highly substituted zones called “hydrophobic zones” and less substituted zones called “hydrophilic zones.” MC has been extensively investigated for biomedical applications as it is water soluble [108] and shows some excellent properties such as film-forming ability, lipid barrier function and low oxygen as well as moisture vapor transmission rate [109]. MC based thermosensitive hydrogel system showed low protein adsorption and cell adhesion [110]. Nevertheless, hydrogel formation of MC between 60 and 80 °C is not suitable for application as injectable hydrogel system and in situ gel formation by MC requires further chemical modification or combining with other thermoresponsive polymers. Grafting of synthetic *N*-isopropylacrylamide (NIPAAm) onto MC combined the thermogelling properties of both materials and the mechanical strength of the hydrogel was also enhanced [111]. Moreover, gel was formed around body temperature within a certain range of MC content in the combined system. However, the thermoresponsiveness of the hydrogel was found to be dependent on the MC concentration in the system. At lower contents of MC, LCST of the hydrogel was decreased, whereas increased MC content in the gel raised the LCST [111]. A thermoresponsive double gel was prepared by incorporating κ- carrageenan and MC mixtures in water and it showed double thermal gel-sol-gel transition upon heating [112]. This system showed a very interesting and reversible thermal behavior as liquid (sol) state of the system was found between the low-temperature and high-temperature gel-state and state of the system could be altered simply by easy temperature tuning [112]. Another injectable thermosensitive hydrogel was prepared from a blend of MC and chitosan (CS) for tissue engineering and it was formed under mild conditions via addition of salts, such as NaCl, Na_3_PO_4_, NaHCO_3_ and glycerophosphate, without applying organic solvent, high temperature or harsh pH [113]. Such blends were liquid at low temperature (~4 °C) and formed gels around body temperature (37 °C) [113]. The thermo-reversible gel system made from MC-pluronic F127 micelle was applied for local and sustained delivery of docetaxel. The combination system was found to release docetaxel for more than 30 days and thus the drug delivery system enhanced the anticancer effect of drug and prolonged it action in comparison to free drug [114]. The hydrogel system made of MC and pluronic F127 showed gel formation around 40 °C and the addition of ammonium sulfate up to 5.0% caused gradual decrease of LCST of the hydrogel close to body temperature of 37 °C. Also, longer gel formation time and low viscosity of the combined system of MC and pluronic F127 were substantially overcome by addition of ammonium sulfate [114].

The transdermal drug delivery system based on thermoresponsive hydrogel of Poloxamer 407 and carboxymethyl cellulose sodium (P407/CMCs) was applied for the treatment of AD with the Chinese herbal medicine (cortex moutan) [51,52]. The hydrophilic CMCs was used to reinforce the porous channels and regulate the physicochemical properties of poloxamer hydrogel. The drug delivery system with cortex moutan was applied in smart textiles for the treatment of AD. P407/CMCs composite thermosensitive hydrogel offered dual-functions of both moisture and drug supply and transdermal drug delivery behavior revealed that P407/CMCs showed desirable percutaneous performance. Also, clinical trials indicated that thermoresponsive hydrogels of P407/CMCs loaded with cortex moutan can deliver moistures to the skin and lessen the symptoms of AD [51,52]. Nevertheless, the drug loading capacity of hydrogel and coating method for uniform distribution of hydrogels on textiles are still required to be improved in order to enhance its applicability for alternative treatment of AD by textile based transdermal therapy. The drug delivery system made of carboxymethyl cellulose/gelatin copolymer hydrogel loaded with lidocaine was applied for transdermal drug delivery [115]. Microneedles and sonophoresis pre-treatment were applied for percutaneous delivery of lidocaine using this hydrogel and this application was considered a viable alternative to conventional percutaneous delivery of drugs by hypodermic needles [115]. Another transdermal drug delivery system made of pH-sensitive hydroxyethyl cellulose/hyaluronic acid complex hydrogel containing isoliquiritigenin was applied for the treatment of skin lesions caused by pH imbalances [116]. The pH responsive hydrogel combined skin compatibility and pH responsiveness of hyaluronic acid and scaffold forming property of hydroxyethyl cellulose to form the hydrogel using different ratio of the components.

### 2.3. Gelatin/Collagen

Gelatin is a natural protein with thermoresponsive properties and is commercially produced from by-products of the meat and leather industries. Gelatin is mainly obtained from animal tissues such as beef bones, cartilage, tendons and pig skin after boiling them. Gelatin, cooked form of collagen is mainly obtained by partial hydrolysis of collagen. Collagen is the major structural protein of connective tissues (tendon, ligament), skin, hair, nails and bones in mammals and makes up to 35% of the protein content in the human body. There are at least sixteen different types of collagen found in the human body and Type 1 is the most abundant and considered to be the strongest type of collagen. Type 1 collagen made up of eosinophilic fibers forms tendons, ligaments, bones and skin and helps in would healing and holding the tissues together. Gelatin is commercially used in food and pharmaceutical industries to get the beneficial amino acids of collagen in the edible form. Gelatin is biodegradable and biocompatible and can accept easy modifications on the amino acid level [117]. Gelatin is versatile due to its intrinsic features that enable the design of different carrier systems, such as hydrogels, micro and nanoparticles and fibers. Gelatin can form biocompatible hydrogels but its biomedical applications are limited due to low mechanical strength which makes additional modifications necessary. The collagen source, the type of hydrolytic treatment used, the extraction method, the amount of thermal denaturation employed and the crosslinking degree of gelatin influence the mechanical properties, swelling behavior, thermal properties and other physiochemical properties [118]. Hydrogels made of an interpenetrating network of gelatin, sodium alginate and 50 wt % of CNCs were applied for cartilage applications and the composite hydrogel cartilage showed modulus clearly higher than that determined for natural cartilage [119]. Hyaluronan-gelatin (HG) hydrogels, along with wood-derived nanofibrillar cellulose, were used for generating a 3D culture environment for HepaRG liver progenitor cells and these are promising materials for hepatic cell culture and tissue engineering [120]. The improved cell morphology, expression and localization of hepatic markers, metabolic activity and vectorial transport were achieved using these materials [119]. Double-network (DN) hydrogels with high mechanical strength was synthesized using gelatin and bacterial cellulose [121]. Gelatin hydrogel crosslinked by microbial transglutaminase exhibits excellent performance in cell adhesion, proliferation and differentiation and the cell migration experiment and subcutaneous implantation confirmed that hydrogels suitable for cell delivery [122]. The hydrogels with high mechanical strength and excellent biocompatibility were developed from chitosan and gelatin for cartilage regeneration and the hydrogels showed designable shapes and special hollow-formed character [123]. The hydrogel made of succinylated gelatin was used as a delivery system for stromal cell-derived factor-1 (SDF-1) and the release profile rate of SDF-1 from the hydrogel was reported to be controlled by changing the water content of the hydrogel during hydrogel preparation [124]. Gelatin methacryloyl (GelMA) hydrogels were used for various biomedical applications, such as tissue engineering, drug and gene delivery, cell signaling and bio-sensing, due to their suitable biological properties and tunable physical characteristics [125].

The aqueous gelatin solution solidifies at temperatures below 25 °C due to the formation of triple helices and a rigid three-dimensional network and it turns to liquid when the temperature is raised above approximately 30 °C as the conformation changes from a helix to the more flexible coil [126]. However, biomedical applications need opposite thermal behavior from the polymer and thereby, gelatin was combined with other polymers to show thermoreversible properties close to body temperature. An injectable thermosensitive hydrogel system was formed from blends of chitosan and gelatin and gel formation from solutions occurred when they were brought into body temperature [127]. Also, chitosan precipitation under basic condition (pH > 6.2) was prevented by addition of gelatin and the hydrogel was solely formed from chitosan/gelatin solution (pH 7) increasing temperature from 4 to 37 °C. The thermoresponsive gel system was biocompatible, biodegradable and adhesive to human tissue and used for the delivery of protein drug bovine serum albumin [127]. NIPAAm was grafted onto gelatin using different graft density and molecular weight of graft chain to produce a series of thermoresponsive extracellular matrix [128]. The matrix showed gelation around body temperature of 37 °C but the biodegradability of the matrix was not reported [128]. Gelatin was blended with silk fibroin to yield a thermoresponsive gel at 37 °C by the presence of β crystals of silk fibroin [129]. Nevertheless, gelation of the protein composites around body temperature (37 °C) caused extensive mass loss due to dissolution and release of gelatin from the mixture, while gelation at 20 °C displayed negligible mass loss of gelatin [129]. The thermoresponsive gel system was developed from chitosan-gelatin-glycerol phosphate to apply as cell carrier for nucleus pulposus regeneration [130]. The gel strength was increased and gelation time was shortened after combining gelatin in the system made of chitosan-glycerol phosphate but the gelation temperature reported (31.3–33.8 °C) was lower than body temperature of 37 °C [130]. The thermoresponsive binary component hydrogel composed of gelatin and monomethoxy poly(ethylene glycol)-poly(d,l-lactide) (MPEG-PDLLA) diblock copolymer was used as a drug delivery system for the antibacterial drug gentamicin sulfate and the thermosensitive hydrogel was found to be capable of gelation within a narrow temperature range between body temperature and room temperature [131]. The combination of gelatin with the MPEG-PDLLA co-polymer changed the thermoresponsive property of gelatin and the copolymer was reported to be hydrolyzed in physiological conditions, indicating its biodegradability when used as a drug carrier [131]. A series of composite hydrogels made of NIPAAm and maleylgelatin with thermoresponsive properties were applied for biomedical applications such as drug delivery and tissue engineering [132]. The combination of maleylgelatin with PNIPAAm improved mechanical properties and the thermodynamic stability of the thermoresponsive hydrogel. With an increase of MAGEL content from 0 to 50%, the composite hydrogel with relatively high water content possessed good compressive strength, tensile strength and stretchability [132].

The hydrogel made of carboxymethyl cellulose/gelatin copolymer loaded with lidocaine was applied as drug delivery system for transdermal drug delivery and microneedles and sonophoresis pre-treatment were applied for percutaneous delivery of drug [115]. The chitosan–gelatin microcapsules loaded with patchouli oil was grafted onto cotton fabric using 2D resin (dimethyloldihydroxyethylene urea, DMDHEU) as a crosslinking reagent [133]. The microcapsules were stable below 190 °C as obtained by the thermal stability analysis and that meant the fabrics finish method was conducted at 160 °C. Chitosan–gelatin microcapsules were formed by way of a complex coacervation process and the antibacterial rate of the fabrics for *Staphylococcus aureus* and *Escherichia coli* were about 65% even after being washed 25 times, suggesting its potential application in many fields such as antibacterial mask, bacteriostatic sheet and health-care clothes [133]. Nevertheless, spherical microcapsules with mean size more than 10 µm reported in this work [133] is not considered very suitable for drug delivery application especially for transdermal treatment as interaction between drug delivery system and cells on skin tissue or any other tissue prefers delivery system having average size less than 1 µm. Automated textile technologies for making fabrics allow simultaneous control over the color pattern and directional mechanical properties and for this reason the concept of composite living fibers (CLFs) was introduced and CLFs were made of a core of load bearing synthetic polymer which was coated by hydrogel layer containing living cells or microparticles [134]. The hydrogel layer was made of alginate/methacrylated gelatin or thermally cross-linkable hydrogels such as collagen and gelatin [134]. This new and innovative textile technology has been utilized for the biofabrication of fibrous scaffolds for various tissue engineering applications [134].

## 3. Synthetic Polymers

### 3.1. Poly(N-isopropylacrylamide)

Thermoresponsive systems based on hydrogels from poly(*N*-isopropylacrylamide) (pNIPAAm) and its copolymers are subject of extensive research and pNIPAAm based hydrogel materials are useful for developing drug delivery systems, sensors and actuators, separation operations in biotechnology, processing of agricultural products [135]. NIPAAm is an acrylamide group monomer used to produce pNIPAAm thermosensitive polymer or co-polymer based hydrogels. pNIPAAm is one of the most intensely studied polymers in reference to biomedical applications due to its LCST which is very close to body temperature and it is capable of showing fast on off switching [136,137]. pNIPAAm assumes a flexible, extended coil conformation in aqueous solutions below the LCST and shows a volume phase transition around 32 °C (LCST) in pure water [138,139]. The polymer becomes hydrophobic at the LCST and the polymer chains seem to collapse prior to aggregation in globular structures and result in changing from coil to globular structure [140]. The thermosensitive hydrogel system based on pNIPAAm is dominated by hydrogen bonding [141]. The copolymerization with a more hydrophobic monomer results in a lower LCST than pNIPAAm [142]. Likewise copolymerization of NIPAAm with a more hydrophilic monomer increases the overall hydrophilicity of the polymer and the stronger polymer-water interactions lead to an increase in the LCST [142]. Recent developments on pNIPAAm-based thermosensitive gels mainly focus on drug delivery applications [143,144,145,146].

Since the LCST of pNIPAAm is close to human body temperature it could be easily adjusted to the physiological range by the copolymerization of NIPAAm with more hydrophilic monomers such as acrylamide [147], *N*-(2-(dimethylamino)ethyl) methacrylamide [148] or poly(ethylene glycol) methacrylamide [149]. The co-networks of NIPAAm with butyl methacrylate (BuMA), p(NIPAAm-co-BuMA) showed zero order drug release profiles and after a burst release of drug from the outer part of the hydrogel, a sustained release was obtained [150]. The drug delivery system made of synthetic polymers should be assessed in terms of its biodegradability and there was no mention of the biodegradability of the system p(NIPAAm-co-BuMA). NIPAAm was grafted onto MC to combine the thermogelling properties of both materials and a lower percentage of MC than pNIPAAm decreased the LCST of the hydrogel but LCST was increased with high MC ratio [111]. Moreover, the addition of MC to NIPAAm polymers enhanced the mechanical strength of the hydrogel [111]. Nevertheless, the biodegradability of cellulose derivative (MC) could be changed after NIPAAm grafting on to it but it was not mentioned in the study about biodegradable nature of thermoresponsive hydrogel made of MC and NIPAAm. The thermoresponsive hydrogel of NIPAAM copolymers with acrylic acid (AA) p(NIPAAm-co-AA) was applied as a cell and drug delivery vehicle [151]. The thermo-reversible hydrogel loaded with chondrocytes and other differentiation materials including TGF β-3, dexamethasone and ascorbate was used as an injectable delivery vehicle for neo-cartilage formation [151]. The copolymers of NIPAAm with propylacrylic acid (PAA) prepared by way of a reversible addition fragmentation chain transfer (RAFT) polymerization method displayed temperature- and pH-sensitive behavior and NIPAAm-co-PAA copolymers displayed tunable properties that could make them useful in a variety of molecular switching and drug delivery applications where responses to small pH changes are relevant [152]. However, the biological function of dual temperature and pH responsive system was not reported and biodegradability/biodegradability of the co-polymer was also not reported in this study. Co-polymer hydrogels developed from pNIPAAm and poly(*N*-butylacrylamide) provided a sustained release of drugs from the film over a considerable time period [153]. 50:50 NIPAAm: poly(*N*-butylacrylamide) in the copolymers could deliver bioactive concentrations of the antimitotic agent colchicine to human vascular cells over an extended period of time and the copolymers did not show any cell toxicity as evidenced by cell viability test [153]. The composite hydrogel materials made of pNIPAAm, nanoparticles and a photo cross-linker—poly(ethylene glycol) diacrylate (PEGDA)—were used for drug delivery applications [154]. Bovine serum albumin (BSA)-loaded pNIPAAm nanoparticles were used to form three-dimensional gel networks by way of a photocuring process using a photo cross-linker, PEGDA and a photoinitiator—Irgacure-2959 (I-2959) [154]. The nanoparticle hydrogel system having thermoresponsive property was also capable of showing in situ photopolymerization to localize at specific location in the body [154]. However, cell toxicity and biodegradability of this hydrogel system were not mentioned in the study. Thermoresponsive hydrogels made from poly(*N*-isopropylacrylamide) (pNIPAAm), cross-linked with poly(ethylene glycol) diacrylate (PEG-DA) were applied for as ocular drug delivery platform for delivering proteins like BSA or immunoglobulin G (IgG) [155]. The hydrogel was reported to be non-toxic and the co-polymer made of NIPAAM and PEG-DA was claimed to be biocompatible [155]. The thermoresponsive hydrogel system showed gelation at (~32 °C) which was lower than body temperature of 37 °C suggesting some limitations in using this as injectable hydrogel system. Thereby, pNIPAAm based hydrogels impede some of their bio-medical applications because of cell toxicity, non-biodegradability and lack of biocompatibility [156]. The drug delivery system was developed from the biodegradable thermo-responsive materials of poly(*N*-isopropylacrylamide-co-*N*,*N*-dimethylacrylamide), which were basically block copolymers made of hydrophobic blocks, namely, biodegradable poly(d,l-lactide), poly(ε-caprolactone), or poly(d,l-lactide-co-ε-caprolactone) and thermo-responsive blocks made of pNIPAAm [157]. The hydrogels comprising both hydrophobic domains and hydrophilic aqueous phase simultaneously encapsulated and delivered hydrophilic drug gemcitabine and hydrophobic drug doxorubicin [157].

pNIPAAm based thermoresponsive hydrogel was tested for various textile applications [158,159]. PNIPAAm-coated cotton fabric showed an extraordinary capacity of collecting water from humid air [158]. The LCST of pNIPAAm coated on to the cotton fabric was influenced by the polymer characteristics including number average molecular weight, poly dispersity index and chain grafting density and also experimental/environmental conditions [158]. The cotton fabric functionalized with a dual-responsive spiropyran–NIPAAm hydrogel formed by a facile controlled polymerization method was reported to be capable of dimensional changes upon irradiation with visible light or a temperature stimulus [160]. A facile and versatile surface-initiated controlled polymerization method (SI-ARGET-ATRP) was applied to form dual-responsive functionalized cotton fabric which showed tremendous potential of absorbing water from air [160]. The cotton fabric coated with pNIPAAm via atom transfer radical polymerization exhibited an excellent transition from superhydrophilicity to superhydrophobicity with changing temperature [161]. The further chemical modification of pNIPAAm improved its transition from superhydrophilicity to superhydrophobicity with changing temperature and this is considered to be helpful for developing thermoresponsive smart textiles [161]. The stimuli-responsive pNIPAAm/chitosan nano hydrogel was applied on cotton fabric as a surface modifying system and the bound nano-hydrogel to the surface of cotton fabrics made them responsive to dual stimuli (temperature and pH) [162]. The nano-hydrogel of chitosan and NIPAAM was made by surfactant free emulsion polymerization and coated on to cotton using 4-butane tetra carboxylic acid (BTCA) as an environmental friendly cross-linking agent. The use of nanohydrogels with BTCA enhanced the water retention capacity of cotton fabric [162]. Temperature sensitive pNIPAAm/polyurethane hydrogel grafted nonwoven fabric achieved antibacterial function against *Staphylococcus aureus* and *Escherichia coli* via chitosan modification [163]. The phase transition temperature of hydrogel grafted onto nonwoven fabrics was about 32 °C and the water absorption of the hydrogel grafted fabrics was responsive to temperature [163]. The thermoresponsive hydrogels made of pNIPAAM were applied to textiles to improve their water retention capacity and enhance their capacity for collecting water from air.

### 3.2. Pluronics or Poloxamers

Pluronics or poloxamers are triblock copolymers of poly(ethylene oxide)-*b*-poly(propylene oxide)-*b*-poly(ethylene oxide) (PEO-PPO-PEO) and they are capable of showing thermoresponsive behavior in aqueous solutions at physiological temperature and pH [15,164,165]. The gelling process of pluronics hydrogels is comprised of two steps: (i) a micellization process in which spherical micelles are formed and (ii) a gelation process in which the stacking of spherical micelles occurs to form gel [15]. The endothermic micellization process of pluronics with temperature rise is driven by a decrease in the polarity of ethylene oxide (EO) and propylene oxide (PO) segments and that results in the entropy gain in water, followed by orientations of monomers to form micelles (hydrophobic effect) [15]. Among various types of pluronics, pluronic F127 (PF127) has gained considerable attention due to its wide range of biomedical applications such as drug and gene delivery [166,167,168], tissue engineering [169,170] and burn wound covering [171]. The PPO segment of PF127 comprises of a hydrophobic core as a microenvironment for incorporation of lipophilic drugs whereas, PEO segment of PF127 prevents the adsorption and aggregation of loaded protein [172]. PF127 based hydrogels can be a good substrate for hematopoietic stem cells, supporting their culture and preservation more than conventional tissue culture dishes [173] even though PF127 hydrogels represent a bio-inert environment due to hydrophilicity and flexibility of PEO chains [174].

Nevertheless, PF127 based thermoresponsive hydrogels are found to have inadequate mechanical strength and stability which make them inappropriate for certain biomedical applications [74,175]. A significant amount of research work was done focusing on improving the mechanical strength of thermoresponsive hydrogels formed by PF127 such as mixed polymeric micelles with pluronic P123 [176], tween 80 [177]; conjugation with gold nanoparticles [178]; and hydrophobically modified with carboxymethyl chitosan [179], alginate [180]; and chain extender introducing hexamethylene diisocyanate [181]. The chemically modified PF127 based thermoresponsive hydrogels find applications in developing advanced drug delivery systems [179,182,183,184]. The dual pH and temperature-responsive hydrogel based on polyurethane, PF127, Erythrosine B showed thermo-sensitive sol-gel phase transition above the critical gelation concentration and the hydrogels not only served as drug carriers but also could be utilized as fluorescence imaging probes in biomedical applications [185]. The gelation temperature was adjusted near the body temperature of 37 °C by optimizing the concentration of the copolymer in water. Erythrosine B (EB) was incorporated to offer a fluorescence property to the hydrogel and the polyurethane-PF127-EB copolymer spontaneously self-assembled into hydrogels with a great number of closely packed micelles [185]. Nevertheless, the cell toxicity and biodegradability of the copolymer were not reported in the study for drug delivery applications. The thermoresponsive hydrogel based on glycol chitosan-PF127 conjugate was applied as drug delivery system for doxorubicin [186] and could partially load superoxide dismutase (SOD). The glycol chitosan-PF127 conjugate (GC-PF127) was produced by adding a carboxyl group to the end of PF127 followed by an amidation reaction between carboxylated PF127 and glycol chitosan [186]. GC-PF127 formed micelles at 25 °C and the micelle solutions GC-PF127 and drug loaded GC-PF127 formed injectable supramolecular hydrogels after the addition of α-cyclodextrin (α-CD) in the micelle solutions [186]. The strength of GC-PF127 hydrogel was also tuned by addition of α-CD. The thermoresponsive-hydrogel microspheres made of chitosan (CS) and PF-127 by the emulsion crosslinking method using glutaraldehyde (GA) as a cross-linker were applied for delivery of anticancer drug, 5-fluorouracil [187]. The average size of microspheres ranging from 110 to 382 µm is not considered suitable for drug delivery applications as oral/intravenous mode of treatment does not prefer drug delivery system of average size greater than 1 µm. The hydrogel matrix made of hyaluronic acid (HA) grafted PF127 (HP) was used for delivery of cisplatin and carboplatin and the hydrogel matrix exhibited a sol–gel transition temperature (28.3 °C) [188]. The drug release rate from HP hydrogel was slower than that from PF127 hydrogel and such thermosensitive hydrogel system was expected to be advantageous as an injectable therapeutic formulation for anticancer treatment [188]. The hydrogel formation at 28.3 °C is not suitable for applying as injectable hydrogel system as in situ gel formation under physiological condition requires body temperature of 37 °C. The locally injectable docetaxel nanocrystals loaded d-alpha tocopheryl polyethylene glycol 1000 succinate-modified PF127 (DOC-NCs-TPGS-PF127) thermo-sensitive hydrogels were prepared to reverse the drug resistance of P-glycoprotein (P-gp)-overexpressing human liver cancer SMMC-7721 cell lines [189]. The modifying agent of PF127 (TPGS) did not show any cytotoxicity against L929 fibroblasts but DOC loaded drug delivery system made of TPGS-PF127 showed cytotoxicity against resistant SMMC7721 cells [189]. The thermoresponsive hydrogel system based on PF127 and Pluronic F68 was applied for ocular drug delivery and the results showed that 20 wt % PF127 offered an attractive ocular formulation that could form a transparent gel in situ under physiological conditions with minimal irritation [190]. It was found that the gel was formed at physiological temperature with 20 wt % of PF127 but the gelation temperature went above physiological range of temperatures in binary formulation made of Pluronic F68 and PF127 [190]. 

For textile applications, PF127 based hydrogels are applied for transdermal drug delivery [51,191]. In the treatment of AD by transdermal therapy, the critical requirement of a drug delivery system to show capability of supplying both moisture and drug to the skin of patients. Wang et al. [51,52,192] developed PF127 based thermoresponsive hydrogels by a “cold method”, through which the loss of herbal medicine was avoided and sufficient moisture was incorporated into the hydrogel. The cortex moutan loaded in the hydrogel showed excellent controlled release and percutaneous diffusional behavior [51]. Moreover, the clinical trials indicated that herbal medicine loaded PF127 based thermoresponsive hydrogel were capable of moisturize skin and relieving the symptoms of AD as well [51,192]. Nevertheless, more standardizations are required to improve the drug loading capacity of PF127 based hydrogel and coating method is still needed to be improvised for uniform distribution of hydrogels on textiles for effective treatment of AD by textile based transdermal therapy. The transdermal studies showed that permeability of the drug through the skin was enhanced with addition of CMCs in the hydrogel formulation [192].

## 4. A comparison of the Different Copolymers with Their Benefits and Disadvantages

Various thermoresponsive polymers (natural and synthetic) were evaluated in terms of their structure, process conditions, properties, and temperature responsiveness, biomedical applications (developing drug delivery systems) and textile based applications for transdermal therapy in Table 1. In this review, the thermoresponsive character of all the polymers was highlighted and PF127 in particular showed great applicability and future prospects to be applied as thermoresponsive hydrogels for textile based transdermal therapies especially for the treatment of AD where the pathogenesis of the disease is still partially unclear and further standardization of textile based transdermal drug supply using thermoresponsive hydrogels will give better answer to the pathogenesis of AD. Nevertheless, it was indicated in this study that all the polymers had some limitations and their derivatives also did not meet all the criteria to become biomedically applicable. Chitosan itself lacks thermoresponsive properties but combining with other thermoresponsive polymers, it formed thermoresponsive hydrogels but in most of the cases, hydrogels were formed at a temperature less than body temperature and in some cases, it did not produce gels of enough mechanical stability. Cellulose derivatives such as MC formed thermoresponsive hydrogels with LCST of 60–80 °C and this is not suitable for application as injectable hydrogel system. Cellulose and their derivatives were combined with other polymers to be applicable as injectable hydrogel system with better drug releasing properties. Gelatin was combined with other polymers to reverse its thermoresponsive properties but in some cases it did not produce hydrogels with enough mechanical stability. It was found form the literature studies that gelatin based hydrogels are normally having LCST around body temperature and it could be advantageous for in situ gel formation especially for drug delivery applications. The thermoresponsive hydrogels made of pNIPAAm and their composites were found capable of in situ gel formation around body temperature and pNIPAAm hydrogels have very effective water absorption capacity. Unlike natural polymers like chitosan, cellulose, gelatin, the biodegradability, cell toxicity and biocompatibility of pNIPAAM based hydrogels were mostly not assured and these were the main reasons for not applying them in real drug delivery systems. PF127 based thermoresponsive hydrogels are the best alternative for developing effective drug delivery systems as they display sol-gel transition near body temperature and the cell toxicity, biodegradability of PF127 based hydrogels has already been proven. There were some PF127 composites which were used for developing thermoresponsive hydrogels with better drug releasing properties and improved applicability. But, cell toxicity, biodegradability and biodegradability of these PF127 based hydrogels were not assured and these missing information impede their real world drug delivery applications.

## 5. Challenges and Potential New Applications/Commercialization

In this context, pH-responsive hydrogels are also very important because of large variations in physiological pH at various body sites in normal and pathological conditions as well [195]. The drug delivery systems made of pH-responsive hydrogels were well applied for cardiovascular treatments [196], gastrointestinal drug delivery [197] and masking the taste- of bitter drugs [198]. Drug delivery systems of pH-responsive hydrogels made from some polymers and their composites like chitosan-poly(ethylene oxide) for amoxicillin, metronidazole [199], gelatin-poly(ethylene oxide) for riboflavin [200], poly(acrylamide-co-maleic acid) for terbinafine hydrochloride [201], poly(2-hydroxyethyl methacrylate) for salicylic acid [202] were successfully applied and some drug delivery systems were also made of dual responsive (pH/temperature) hydrogels from poly(*N*-isopropyl acrylamide-co-butyl methacrylate-co-acrylic acid for delivery of calcitonin [203] and *N*-acryloylglycine methyl ester (NAGME), *N*-acryloylglycine ethyl ester (NAGEE) and acrylic acid (AAc) for caffeine [204]. pH/temperature responsive hydrogels as drug delivery systems for textile based transdermal therapies could be a breakthrough in the treatment of AD as it can more effectively fight against the pathogenesis of this skin disease and the schematic representation of drug loaded dual (pH/temperature) responsive hydrogel for textile based transdermal therapy was given in Figure 3. The dual responsive (pH/temperature) hydrogels with high drug loading and active under physiological condition (37 °C and pH 7) are being developed, characterized and applied on textile fabrics to meet all the requirements for fighting against the pathogenesis related to AD and to develop an effective treatment method of AD.

## 6. Conclusions

A wide range of hydrogels from various natural and synthetic polymers is being applied for various biomedical applications and a significant progress has been achieved through product formulation, scaling and shaping up their properties and enhancing functionality and applicability of hydrogels. Moreover, resemblance of hydrogels to living tissue opens up many biomedical applications including drug delivery, tissue engineering, wound dressings and manufacturing contact lenses. Thermoresponsive hydrogels are a special class of hydrogels which can use small or moderate temperature change as a trigger for sol-gel transition and at body temperature injectable thermoresponsive polymers can from gel from their aqueous solutions. In this review, various thermoresponsive hydrogels made of natural polymers such as chitosan, cellulose, gelatin and synthetic polymers—namely pluronics (PF127) and pNIPPam—were considered and the properties and biomedical applications of thermoresponsive hydrogels were mentioned. A special insight was given to textile applications of hydrogels made by these polymers for textile based transdermal therapies and their thermoresponsive hydrogels were mainly highlighted in this review for transdermal treatment using textiles. Future progress is focusing on development and applications of dual (pH/temperature) responsive hydrogels for effective textile based transdermal therapies.

## Figures and Tables

**Table 1:** An overall view of natural and synthetic polymers used in thermoresponsive hydrogels for drug delivery and transdermal therapy.

Natural Polymers	Structure	Process Condition	Properties and Temperature Responsiveness	Biomedical Applications (Drug Delivery System)	Textile Based Applications for Transdermal Therapy
*Chitosan*	d-glucosamine units and trace amount of *N*-acetyl-d-glucosamine	Exoskeleton of crustacean, from chitin by alkaline deacetylation	Carbohydrate biopolymer, biodegradable, biocompatible and effectively showing thermoresponsive properties after modification/conjugation in the form of hydrogels with gel formation at body temperature [15]	Thermosensitive hydrogels using chitosan, hyaluronic acid and *N*-isopropylacrylamide (NIPAAm) for analgesic drug nalbuphine [85]	Textile based transdermal therapy was done using microcapsules of chitosan and alginate loaded with traditional Chinese medicines (PentaHerbs formula and cortex moutan) [93,94]
*Cellulose*	d-glucopyranose units	Primary cell wall of green plants and many varieties of algae	Natural polysaccharide and various cellulose derivatives like methyl cellulose (MC) forming thermoresponsive hydrogels at 60–80 °C	Thermoresponsive hydrogel system made from MC-pluronic micelle for anticancer drug docetaxel [114]	Textile based transdermal drug delivery system was developed from thermoresponsive hydrogel of PF127 and carboxymethyl cellulose sodium loaded with the Chinese herbal medicine (cortex moutan) [51,52]
*Gelatin*	Amino acids (rich in hydroxyproline proline, glycine)	Animal tissues such as beef bones, cartilage, tendons and pork skin by boiling	Natural polymer made of amino acids and hydrogels made of gelatin derivative show thermoresponsive properties with sol-gel transition at body temperature	Thermoresponsive hydrogel made of gelatin and monomethoxy poly(ethylene glycol)-poly(d,l-lactide) (MPEG-PDLLA) for antibacterial drug gentamicin sulfate [131]	Hydrogel from carboxymethyl cellulose/gelatin copolymer loaded with lidocaine was applied as drug delivery system for transdermal drug delivery [115]
Synthetic Polymers	Structural units	Process condition	Properties and temperature responsiveness	Biomedical applications (Drug delivery system)	Textile based applications for transdermal therapy
*pNIPAAM*	*N*-isopropylacrylamide units	Synthesized from commercially available *N*-isopropylacrylamide via free-radical polymerization [193]	Synthetic polymer with intrinsic thermoresponsive properties with sol-gel transition at body temperature. Derivatives of polymer are capable of forming thermoresponsive hydrogels with better mechanical properties	Thermoresponsive poly(*N*-isopropylacrylamide-co-butyl methacrylate) hydrogel for drug indomethacin [150]	-
*PF127*	Triblock copolymer, central hydrophobic block of polypropylene glycol flanked by two hydrophilic blocks of polyethylene glycol	Synthetized by condensation of ethylene oxide and propylene oxide [194]	Amphiphilic synthetic polymer with intrinsic thermoresponsive properties and can form hydrogels in situ, effectively used as injectable polymer for drug delivery applications	Thermoresponsive hydrogel made of polyurethane-PF127- Erythrosine B for anticancer drug doxorubicin [185]	Thermoresponsive hydrogel from PF127 and carboxymethyl cellulose sodium loaded with the Chinese herbal medicine (cortex moutan) was applied for textile based transdermal drug delivery system [51,52]
